# Vaccination for Patients Receiving Dialysis

**DOI:** 10.1016/j.xkme.2023.100775

**Published:** 2023-12-09

**Authors:** Ramin Sam, Laura Rankin, Ifeoma Ulasi, Luc Frantzen, Dorothea Nitsch, David Henner, Donald Molony, John Wagner, Jing Chen, Sanjay Kumar Agarwal, Andrew Howard, Ralph Atkinson, Daniel Landry, Stephen O. Pastan, Kamyar Kalantar-Zadeh

**Affiliations:** 1 Division of Nephrology, Zuckerberg San Francisco General Hospital, University of California, San Francisco; 2 Kidney Specialists of Central Oklahoma, Oklahoma City, Oklahoma; 3 Division of Nephrology, University of Nigeria, Enugu, Nigeria; 4 College of Medicine, University of Nigeria, Ituku-Ozalla Campus, Enugu, Nigeria; 5 Service de Nephrologie, Hopital Saint Joseph, Marseilles, France; 6 Department of Epidemiology, London School of Hygiene and Tropical Medicine, London, United Kingdom; 7 Department of Non-communicable Disease Epidemiology, London School of Hygiene and Tropical Medicine, London, United Kingdom; 8 Division of Nephrology, Berkshire Medical Center, Pittsfield, Massachusetts; 9 Division of Nephrology, University of Texas McGovern Medical School, Houston, Texas; 10 Division of Renal Diseases and Hypertension, McGovern Medical School, University of Texas Health, Houston, Texas; 11 Division of Nephrology, New York City Health + Hospitals/Kings County, Brooklyn, New York; 12 Division of Nephrology, Huashan Hospital, Fudan University, Shanghai, China; 13 Division of Nephrology, All India Institute of Medical Sciences, New Delhi, India; 14 Nephrology and Renal Transplant Medicine, Marengo Asia Hospital, Gurugram and Faridabad, Haryana, India; 15 Metropolitan Nephrology Associates PC, Clinton, Maryland; 16 Nephrology Associates, P.C., Nashville, Tennessee; 17 Division of Nephrology, University of Massachusetts Chan Medical School-Baystate, Springfield, Massachusetts; 18 Division of Nephrology, Emory University School of Medicine, Atlanta, Georgia; 19 Renal Division, Department of Medicine, Emory University School of Medicine, Atlanta, Georgia; 20 Division of Nephrology, University of California, School of Medicine, Los Angeles, California

**Keywords:** Dialysis, vaccination, SARS-CoV2, influenza, pneumococcus

## Abstract

Vaccinating patients receiving dialysis may prevent morbidity and mortality in this vulnerable population. The National Forum of End-Stage Renal Disease Networks (the Forum) published a revised vaccination toolkit in 2021 to update evidence and recommendations on vaccination for patients receiving dialysis. Significant changes in the last 10 years include more data supporting the use of a high-dose influenza vaccine, the introduction of the Heplisav-B vaccine for hepatitis B, and changes in pneumococcal vaccines, including the approval of the PCV15 and PCV20 to replace the PCV13 and PPSV23 vaccines. Additional key items include the introduction of vaccines against severe acute respiratory syndrome coronavirus 2, the virus that causes coronavirus disease 2019 (COVID-19), and a new vaccine to prevent respiratory syncytial virus disease. Historically, influenza and pneumococcal vaccinations were routinely administered by dialysis facilities, and because of possible risks of hematogenous spread of hepatitis B, dialysis providers often have detailed hepatitis B vaccine protocols. In March 2021, COVID-19 vaccines were made available for dialysis facilities to administer, although with the end of the public health emergency, vaccine policies by dialysis facilities against COVID-19 remains uncertain. The respiratory syncytial virus vaccine was authorized in 2023, and how dialysis facilities will approach this vaccine also remains uncertain. This review summarizes the Forum’s vaccination toolkit and discusses the role of the dialysis facility in vaccinating patients to reduce the risk of severe infections.

Vaccinating patients receiving maintenance dialysis is crucial to reducing morbidity and mortality in this vulnerable population. Specifically, patients receiving dialysis are more vulnerable to complications from an infection because of the high prevalence of immune compromise. Additionally, patients receiving dialysis, particularly those being treated with in-center hemodialysis, are a mandatory congregate population, meaning that they cannot isolate when ill and therefore increase the risk of transmission of an infection to others.

Ideally, vaccination should be given in the dialysis units because patients receiving dialysis spend a lot of time commuting to the dialysis center and dialyzing there, frequently feeling unable to keep many other medical appointments. Hence, administering the vaccines during dialysis will improve adherence. Surprisingly, there are little data on the effectiveness of increasing adherence by vaccinating patients in the dialysis clinic. To the best of our knowledge, only 1 study found that vaccination for coronavirus disease 2019 (COVID-19) in the dialysis clinic reduced disparities in the vaccination rate among patients receiving dialysis.^1^ Clearly, it would be logical to conclude that the practice of vaccination in the dialysis clinic will lead to better vaccination coverage, even without literature supporting it. Another advantage would be that dialysis centers are more likely to have a robust vaccination record.

The advisory committee on immunization practices (ACIP), recently updated in 2022, has stressed the importance of 2 vaccines (hep B and pneumonia) that are recommended for patients receiving dialysis.^2^ The Forum of End-Stage Renal Disease (ESRD) Networks also published a vaccination toolkit online.^3^ The common vaccines for influenza, pneumococcal pneumonia, and hepatitis B are summarized in Table 1.

**Table 1:** Common Vaccines for Influenza, Pneumococcal Pneumonia, and Hepatitis B

Vaccine	Brand Name	Disease	Year Approved by FDA	Indication	Effectiveness
**PPSV23**	Pneumovax (Merck)	Pneumococcus	1983		
**PCV13**	Prevnar 13 (Pfizer)	Pneumococcus	2011		Covers additional serotypes
**PCV15**	Vaxneuvance (Merck)	Pneumococcus	2021		
**PCV20**	Prevnar 20 (Pfizer)	Pneumococcus	2021		
	Fluzone Quadrivalent (Sanofi)	Influenza	2013		
	Fluzone high dose Quadrivalent (Sanofi)	Influenza	2014	Age > 65 y	More effective because it has 4 times the antigen∗
**Hepatitis B Vaccine (Recombinant)**	Recombivax (Merck)	Hepatitis B	1986		
**Hepatitis B Vaccine (Recombinant)**	Engerix-B (Glaxo-Smith-Kline)	Hepatitis B	1989		
**HepB-CpG**	Heplisav-B (Dynavax)	Hepatitis B	2017		More effective than other Hep B vaccines

### Pneumococcal Vaccination

In 1983, a 23-valent polysaccharide vaccine (PPSV23, Pneumovax 23) was licensed to minimize the risk of pneumococcal infections. Until recently, it was recommended that most adults over 65 years receive 1 dose.

In 2011, a second pneumococcal vaccine became widely available to further minimize the risk of pneumococcal disease. Polysaccharide conjugate vaccine 13 (PCV13 or Prevnar 13) is a conjugate vaccine that covers most of the pneumococcal serotypes that cause this invasive disease. Two newer pneumococcal conjugate vaccines, PCV15 (Vaxneuvance) and PCV20 (Prevnar 20), approved in 2021, are now part of the recommendations, leading to the removal of PCV13 wherever the newer vaccines are available. Eventually, pneumococcal vaccination would likely involve just 1 dose of PCV20 in a person’s lifetime (Fig 1), as mentioned below:

• For patients who have not received any previous pneumococcal vaccine, the ACIP recommends 1 dose of PCV20 (which completes their vaccinations) OR PCV15 plus 1 dose of PPSV23 at least 1 year later, with a possible minimum interval of 8 weeks for immunocompromised patients (this does not include dialysis by itself unless the patient receiving dialysis is receiving immunosuppressive medications). Not all dialysis units may have these newer vaccines available. and they should continue to use PCV13 until the newer vaccines become available. For patients who only received PPSV23 previously, the ACIP recommends a single dose of PCV15 or PCV20 at least 1 year after PPSV23.
• For patients who received PCV13 previously, the recommendation is PPSV23 at least 8 weeks later and up to 3 total doses (2 doses before age of 65 ≥ 5 years apart, and the last dose after age of 65 > 5 years after the previous dose). Alternatively, 1 dose of PCV20 may be administered to complete the vaccinations.

**Figure 1:**
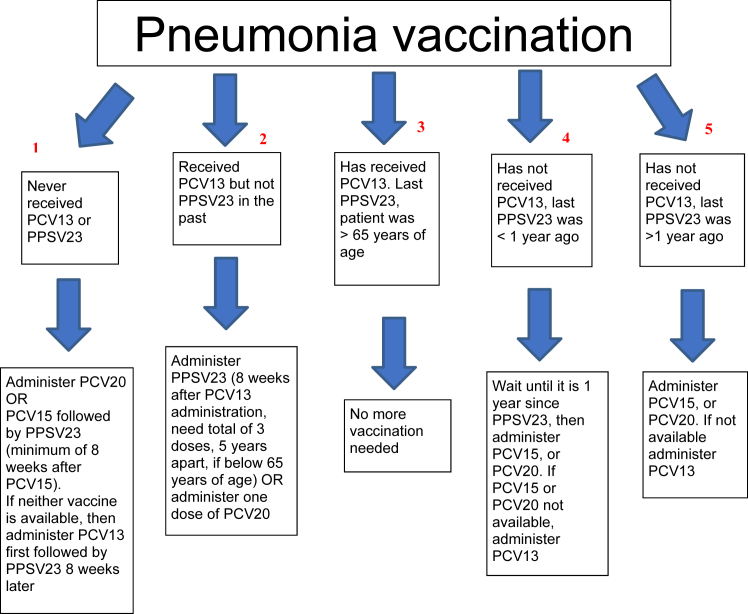
Pneumonia vaccine protocol. To undergo pneumonia vaccination we need a record of the previous vaccinations that patients have received. Following to be noted:1. Prevnar 15 or 20 vaccine is only given once in lifetime of the patient.2. PPSV23 vaccine can be given as many as 3 times in the life of the patient.3. One dose of Prevnar 20 will complete a patient’s pneumonia vaccination.4. One dose of Prevnar 15 will require one additional dose of PPSV23 to complete vaccination series.5. Prevnar 15 or 20 vaccine cannot be given up to a year after receiving PPSV23 vaccine.6. PPSV23 can be given as early as 8 weeks after giving Prevnar 15.7. PPSV23 is readministered 5 years after giving first dose in patients less than 65 year of age and then again after age of 65, assuming at least 5 years elapsed since the last dose.8. After age 65, only 1 dose of PPSV23 is necessary.

No further vaccination is needed for patients who already completed the PCV13 and PPSV23 series. For further information, see pneumococcal vaccine timing for adults: https://www.cdc.gov/vaccines/vpd/pneumo/downloads/pneumo-vaccine-timing.pdf

### Influenza Vaccination

Influenza infection is associated with significant morbidity and mortality in the general population, especially the elderly. The consequences of infection in patients receiving dialysis are likely even more alarming. In a study of patients receiving hemodialysis with diabetes, 17% of patients not vaccinated were diagnosed with influenza, compared with only 6.3% among vaccinated patients (likely some influenza infections in both groups were not diagnosed by testing).^4^

There is widespread consensus that all patients receiving dialysis should be vaccinated against influenza annually. However, from 2014 to 2017, only 72.1% of patients receiving dialysis were vaccinated.^5^ Questions about influenza vaccination in patients receiving dialysis are as follows:

1. Should all patients receiving dialysis receive a high-dose influenza vaccination, or only those >65 years old?
2. When is the best time to vaccinate patients receiving dialysis?

In 2014, the US Food and Drug Administration (FDA) approved the Fluzone high-dose quadrivalent influenza vaccine (Sanofi Pharmaceutical) for people > 65, which contains 4× greater antigen and elicits a higher antibody response; however, both available influenza vaccines are quadrivalent (ie, 4 targeted variants of influenza, 2A and 2B forms). Currently, the high-dose vaccine is recommended only for patients older than 65 years, regardless of being immunocompromised. In 2018, a study examining the high-dose vaccine in patients receiving dialysis during the 2015-2016 and 2016-2017 influenza seasons demonstrated a decrease in hospitalizations in 2016-2017 but not in 2015-2016 compared with the regular dose vaccine. No improvement in death rates in either influenza season was demonstrated.^6^ Another study showed more promising results with the high-dose quadrivalent vaccine.^7^ An analysis of USRDS data between the 2010 and 2015 influenza seasons found the risk for mortality, hospitalizations due to influenza or pneumonia, and influenza-like illness did not differ between patients who received the high-dose vaccine versus the standard-dose vaccine.^8^ However, the study may have been biased because most of the patients receiving the high-dose vaccine were older. An analysis of the younger than 65 years group still did not show a benefit for the higher dose vaccine, but the number of patients in this sample may have been low, and it is likely that the standard dose group may still have been younger and with fewer comorbid conditions than the high dose group because this was not a prospective study.

In most years, the influenza season is from October to May, with influenza typically peaking in December until February. The Center for Disease Control (CDC) recommends administering the influenza vaccine in the United States before the end of October. Data suggest that giving the vaccine earlier (ie, August) will result in the waning of its effectiveness before the end of the influenza season. At least 1 study found that administering the vaccine from late October to mid-November was ideal^9^; however, there have been reports of influenza infections as early as October in some years. For populations in the southern hemisphere, the recommended months for administration should be adjusted. All patients should receive influenza vaccination, even late in the influenza season.

Quadrivalent inactivated influenza vaccines (IIV4), some of which are egg-based, make egg allergy a contraindication (although it may be safe to give egg-containing vaccines to most patients with egg allergies) (https://www.cdc.gov/flu/prevent/egg-allergies.htm). By contrast, the quadrivalent recombinant influenza vaccine (RIV4) is produced in an insect cell line with no viruses or eggs. Quadrivalent live attenuated influenza vaccine (LAIV4) is approved for ages 2-49 years and is given intranasally. It is contraindicated for immunocompromised patients, their close contacts and caregivers, pregnant women, and asthmatic patients; however, many patients receiving dialysis can receive this vaccine despite some degree of immunosuppression from the kidney disease.

More detailed CDC and World Health Organization (WHO) recommendations for influenza vaccination are available at https://www.cdc.gov/flu/professionals/acip/summary/summary-recommendations.htm https://www.who.int/teams/immunization-vaccines-and-biologicals/policies/who-recommendations-for-routine-immunization---summary-tables

### Hepatitis B Vaccination

Three hepatitis B vaccine formulations are available. Recombivax HB (Merck) and Engerix-B (Glaxo-Smith-Kline) are manufactured from viral proteins generated in yeast through recombinant DNA technology and have an aluminum adjuvant. The third vaccine, HepB-CpG; sold as Heplisav-B by Dynavax, is the newest of the 3 and will be discussed later. For the 2 original vaccines, 3 doses are recommended for the general population, but for patients receiving dialysis, higher vaccine doses (40 mcg vs 10 mcg for Recombivax HB vaccine) or additional doses are recommended per vaccination series (3 doses of Recombivax HB and 4 of Engerix-B) because patients receiving dialysis have subnormal immune responses to these vaccines.^10^ A second series is indicated if seroconversion does not occur after the first vaccine series. If a second full hepatitis B vaccine series fails to induce an HbsAb titer > 10 mIU/mL, the patient is considered a nonresponder, and no further vaccine doses are indicated. The overall response rate to vaccination in the dialysis population with these regimens is, at best, 70%. New evidence supports administering a series of the newer vaccine, Heplisav-B, if the patient has not responded to the older vaccines.

Once seroconversion occurs, patients should have annual surface antigen and quantitative HbsAb testing. A booster vaccine should be administered when antibody titers drop below 10 mIU/mL. Not checking HepBsAg in recently vaccinated patients is recommended because antigen levels may persist up to 2 weeks after vaccination, leading to false-positive results, possible unnecessary isolation, and repeat testing.^11^ We recommend not to isolate these patients because it may result in more harm than benefit. Given the suboptimal seroconversion response among patients receiving dialysis, it is recommended to immunize previously unvaccinated advanced patients with chronic kidney disease to hepatitis B and pneumococcal disease before dialysis initiation.

In 2017, a third recombinant hepatitis B vaccine became available in the United States (HepB-CpG; sold as Heplisav-B by Dynavax), and in 2021 it received approval by the European Commission Marketing Authorization. Heplisav-B contains a novel immunostimulatory adjuvant and requires administration of only 2 doses (1 month apart) versus 3 doses required for Recombivax or Engerix. The CDC and ACIP recommendations suggest serologic testing 1-2 months after the second dose for patients receiving hemodialysis. If anti-HBs titers are <10 mIU/mL, then another 2 doses of HepB-CpG should be given, followed by anti-HBs testing (> 2 complete hepatitis B vaccine series are not recommended) (https://www.cdc.gov/mmwr/volumes/67/wr/mm6715a5.htm).

A preliminary study showed a higher antibody response rate with 3 doses of HepB-CpG compared with 4 doses of Engerix-B in patients with chronic kidney disease.^12^ A study of these patients at a mean follow up of 2.5 years indicated that titers were higher with 3 doses of HepB-CpG than Engerix. A more recent study of 119 patients receiving hemodialysis, 75 of whom completed the protocol, tested the efficacy of 4 doses of HepB-CpG (20 μg) at 0, 4, 8, and 16 weeks. Anti-HBs testing was performed each week of vaccine administration and at week 20. Titers > 10 mlU/mL were found in 56.8% after 2 doses, 78.7% after 3 doses, and 89.3% after 4 doses.^13^ By comparison, antibody titers >10 mlU/mL were found in 73.7% of patients receiving 4 doses of 40 mcg Engerix and 64.3% in patients receiving 3 doses of 40 mcg Recombivax. At least one large dialysis organization has used this article to develop a 4-dose protocol for Heplisav-B. As noted above, the CDC and ACIP guidelines suggest administering 2 doses followed by serologic testing and repeating the 2-dose series if needed.

Over the last year, many dialysis providers in the US and likely other countries have moved to vaccinate their patients according to the above schedule with the HepB-CpG vaccine. HepB-CpG vaccine may soon replace other hepatitis B vaccines in dialysis units worldwide. Fig 2 demonstrates an algorithm for hepatitis B vaccination.

**Figure 2:**
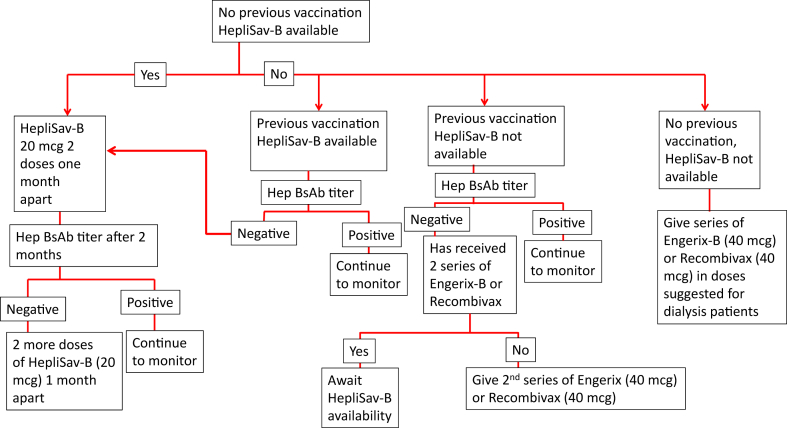
Hepatitis B vaccine protocol.

### Severe acute respiratory syndrome coronavirus 2 vaccination

As of mid-2023, there were 9 vaccines approved for use by the WHO against COVID-19: ChAdOx1-S (Astra-Zeneca), BBV152 (Bharat Biotech), Ad26.CoV2.S (Johnson and Johnson), BNT162b2 (Pfizer), mRNA-1273 (Moderna), NVX-CoV2373 (Novavax manufactured under 2 different brand names in India and Europe), VLA2001 (Valneva), BBIBP-CorV (Sinopharm), and CoronaVac (Sinovac) (Table 2).^14^, ^15^, ^16^ Two of the above vaccines (BNT162b2 and mRNA-1273) are mRNA vaccines, 3 are inactivated virus vaccines (BBIBP-CorV, CoronaVac, and BBV152), 2 are adenovirus vector-based vaccines (ChAdOx1-S and Ad26.CoV2.S), 1 is inactivated, adjuvanted whole virus vaccine (VLA2001) and 1 is adjuvanted protein vaccine (NVX-CoV2373). Four of the vaccines (Ad26.CoV2.S, BNT162b2, mRNA-1273, and NVX-CoV2373) are approved by the US FDA for, at a minimum, emergency use. As of August 2021 and January 2022, respectively, the BNT162b2 (aged 16 years and older) and mRNA-1273 (aged 18 years and older) vaccines have received full FDA approval and have emergency use authorization for children 6 months and older in the United States. The original regimen for all of these vaccines was 2 doses spaced 2-4 weeks apart, except for Ad26. The CoV2.S vaccine (currently recommended only for people who cannot take or refuse the mRNA vaccines) was 1 dose. These vaccines are recommended for ages > 12, 3 months after the last dose of the COVID-19 vaccine (after the primary series) or infection with the virus. Because the Omicron variant has many more mutations^17^ than the original strain, as opposed to the Delta variant, which reported 4 mutations, the original vaccines may not be as protective against infection.^15^ Even the original vaccines seem to still protect against severe disease. Newer bivalent versions of the BNT162b2 and mRNA-1273 vaccines, first available in fall 2022, elicit higher antibody titers against the newer Omicron variants of the virus.^16^ Most recently, an updated COVID-19 vaccine has become available for the fall and winter of 2023-2024.

**Table 2:** Vaccines Against Severe Acute Respiratory Syndrome Coronavirus 2

Vaccine	Company Making the Vaccine	Approved by WHO	Approved by US FDA	Age Approved	Original Number of Doses Recommended
**ChAdOx1-S**	Astra-Zeneca	Yes	No	>18 y	2
**Ad26.CoV2.S**	Johnson and Johnson	Yes	Emergency use approval for patients not eligible for mRNA vaccine	>18 y	1
**BNT162b2**	Pfizer	Yes	Full approval for >5 y of age	>6 mo of age to 5 y (EUA)	2
**mRNA-1273**	Moderna	Yes	Full approval for >18 y of age	>6 mo of age to 17 y (EUA)	2
**BBIBP-CorV**	Sinopharm	Yes	No	>18 y	2
**CoronaVac**	Sinovac	Yes	No	>18 y	2
**NVX-CoV2373**	Novavax	Yes	No	>18 y	2
**Gam-COVID-Vac**	Sputnik V	No	No		2

Abbreviations: EUA, emergency use authorization, FDA, Food and Drug Administration; WHO, World Health Organization.

As per the FDA, testing for antibody levels should not be used to evaluate a person’s level of immunity from COVID-19. Many tests are under emergency use authorization from the FDA; none are truly quantitative. In addition, the level of protection from infection is not known. The discussion below should therefore be viewed within this context.

As a consequence of natural infection, a mean antispike antibody titer of 212 UA/mL (551 BAU/mL) is seen in patients receiving dialysis a year after severe COVID-19 infection.^18^ After 2 doses of BNT162b2 vaccine, 82% of patients receiving dialysis reported neutralizing antibodies compared with 100% of healthy controls (with a median percent inhibition of 51% in patients receiving dialysis and 98% in controls).^19^ The antibody response rate in patients receiving transplant seems to be much lower than that of patients receiving dialysis.^20^ Overall, patients receiving dialysis do not seem to be as immunocompromised as patients receiving immunosuppression for organ transplants, but they are more immunocompromised than patients with many other chronic diseases (demonstrated by the fact that the response for instance to hepatitis B vaccination is lower in patients receiving dialysis compared with patients with many other chronic diseases). In 1 study, adequate antibody response was seen in 92% of patients receiving peritoneal dialysis, 84% of patients receiving hemodialysis, and only 26% of patients receiving kidney transplant after 2 doses of COVID vaccines. This is especially true if the patient receives B cell depleting therapy such as rituximab or belatacept.^21^ However, preliminary data indicate that the immune response to natural infection may be similar in patients receiving kidney transplant compared with the response in normal controls, which may be a reflection of more severe illness.^22^

Previous COVID-19 infection clearly augments the antibody response. In 1 study of 186 patients receiving hemodialysis, the antibody titers were clearly higher in this subgroup of 38 patients with previous COVID infection.^23^ Among patients with past history of COVID infection, all 38 patients were responders to 2 vaccine doses, 97% at maximum titers. Of the patients with no history of COVID infection, 86% were responders with less than 70% at maximum titer.

In a study, the mRNA-1273 vaccinated patients reported 2.98-fold higher antibody titer than those vaccinated with the BNT162b2 vaccine.^24^ A study on ChAdOx1-S vaccine found the vaccine provided suboptimal antibody protection in patients with COVID-19 infection from variants of concern if they did not have previous COVID-19 infection.^25^ Finally, a study from Thailand on Coronavac (Sinovac Biotech) vaccine in 60 Chinese patients receiving dialysis and 30 healthy controls found an anti-RBD antibody titer of 590 AU/mL in patients receiving dialysis and 1,767 in healthy controls.^26^ Another study from Chile compared 81 patients receiving hemodialysis who received BNT162b2 vaccine with 127 patients receiving hemodialysis who received the CoronaVac vaccine.^27^ The humoral response to the BNT162b2 was superior to that of the CoronaVac vaccine with 98.8% of patients in the BNT162b2 vaccine group having an adequate antibody titer at 4 months after the booster shot as opposed to 86.6% in the CoronaVac vaccinated group.^27^ In the general population, mRNA vaccines are shown to elicit major short-term antibody responses which wane over 3-6 months; these data are lacking in patients receiving dialysis.^15^ However, the adenovirus vector vaccines elicit a much lower initial antibody response, but this response lasts longer, up to at least 8 months.

A multicenter observational cohort study of 1,323 patients receiving dialysis in London having experienced COVID-19 infection found a decrease in hospitalizations by 75% and mortality by 88% in those having previously completed a 2-dose vaccination with BNT162b2 or AZD1222 compared with unvaccinated dialysis patients.^28^ The above results have been duplicated in other studies,^29^^,^^30^ with 1 large observational study from a large dialysis organization showing more than twice the mortality rate from COVID in unvaccinated patients receiving dialysis compared with those who were vaccinated.^17^

It has clearly been demonstrated that antibody titers wane over time in the general population, an effect that may be even more pronounced in patients receiving dialysis. Davidovic et al^31^ found that adequate antibody seroconversion rates against COVID-19 in patients receiving hemodialysis decline from 97.9% at baseline to 65.8% at 6 months. A third dose of vaccines has been shown to increase the serologic response^32^, ^33^, ^34^ in addition to lowering the incidence of severe COVID illness.^35^^,^^36^ A fourth dose of the vaccine also seems to increase the humoral response but possibly not as helpful in patients who already have low antibody titers.^37^^,^^38^ A study found a lower risk of severe COVID illness after 4 doses compared with 3 doses of the BNT162b2 vaccine.^39^ It seems patients receiving dialysis who already have received the Ad26.COV2.S vaccine have a higher antibody response if they receive the booster dose with mRNA vaccine compared with receiving the booster with additional Ad26.COV2.S vaccine.^40^ The bivalent mRNA booster vaccines also benefits patients receiving dialysis, especially those who have not had previous breakthrough infection.^41^ Whether patients receiving dialysis should receive severe acute respiratory syndrome coronavirus 2 (SARS-CoV-2) vaccines yearly with the general population or every 6 months because of being immunocompromised is still a matter of debate, but there are preliminary data reporting that every 6 month administration may be beneficial.^42^ Our recommendation is for all patients receiving dialysis to receive a dose of SARS-CoV-2 current recommended mRNA vaccine every 6 months.

An interesting study found that the humoral response to hepatitis B vaccination may improve in patients receiving dialysis after administration of SARS-CoV-2 vaccine.^43^ In this study, 6 out of 16 nonresponder patients reported adequate hepatitis B surface antibody titers after a booster dose shortly after receiving the SARS-CoV-2 vaccine (all received the Energix vaccine). Further, more definite studies would be helpful to determine if there is an optimal period between administrations of the different vaccines.

COVID-19 vaccination may be associated with flu-like symptoms, such as fevers, chills, malaise and myalgias within the first 24-48 hours in one-third to half of patients receiving dialysis who receive mRNA vaccines.^44^^,^^45^ The Forum of ESRD Networks (and other organizations) have created educational posters and flyers to help patients make informed decisions: https://esrdnetworks.org/resources-news/covid-19-information-resources/resources-for-kidney-patients/

### RSV Vaccination

One of the newest vaccines to become available is the vaccine for respiratory syncytial virus, which can cause disease not only in the pediatric group but can be severe for adults over the age of 65. As of May of 2023, 2 vaccines were approved by the FDA for adults older than 60 years.^46^ According to the CDC website, individual over 60 years who have chronic medical conditions such as kidney disease should consider getting vaccinated. The 2 available vaccines are: RSV Pre F3 (Arexvy), made by Galaxo Smith Kline (against RSV-A), and RSV Pre F (Abrysvo), made by Pfizer (against RSV-A and B). Both are recombinant RSV F protein antigen vaccines. A study of over 34,000 individuals showed the RSV PreF vaccine to have an efficacy of 67% in preventing lower respiratory tract disease and 62% efficacy for preventing RSV-associated acute respiratory illness.^47^ Another study of RSVPreF3 vaccine on over 24,000 people, found this vaccine to be 94% effective in preventing severe RSV-related lower respiratory tract disease and 72% effective in preventing RSV infection in general.^48^ At this point, it seems the vaccine has some residual efficacy, even 2 years after administration. Consistent with CDC recommendation here, it is recommended that patients receiving dialysis over the age of 60 seriously consider and receive an RSV vaccine. The optimal frequency of recurrent administration of the vaccine is yet to be established but likely to be at least every other year. Again, it would be preferable if this vaccine can also be given in the dialysis unit to optimize adherence.

### Other Vaccinations Not Routinely Given During Dialysis

Recommendations for the below-mentioned vaccines in the dialysis population are the same as those in the general population, with most not having been studied extensively in patients receiving dialysis.^3^

• Herpes zoster: The 2 vaccines against herpes zoster include a recombinant zoster vaccine (RZV) and a live zoster vaccine (ZVL). RZV is recommended for patients aged older than 50 years and consists of a series (at least 2 months apart).^49^ The RZV was introduced to the market after ZVL was routinely given, but it has been shown to be more efficacious. As a result, ZVL has been removed from the US market. Patients experiencing an active zoster infection should have their infection cleared before being vaccinated (some have suggested waiting as long as 1 year). If a patient has received ZVL in the past, it is still suggested to vaccinate them with a full course of RZV (given its superior efficacy and duration of immunity).
• Tetanus, diphtheria, and acellular pertussis (Tdap): There are currently 2 available vaccines for adults (Adacel and Boostrix, both Tdap) given as 0.5 mL intramuscular injections.^50^ In addition, there are 2 vaccines intended for infants and children (Daptacel and Infanrix, both DTaP). For the most part, adult vaccines are once in a lifetime single injection with the caveat that pregnant patients should receive an extra dose with each pregnancy (repeat vaccinations are in general not harmful but not indicated except a second dose of Adacel after > 8 years from the initial dose may be given routinely). Pertussis is a highly contagious disease, causing prolonged cough, which used to be seen only in children during the prevaccination era but now is not uncommon in adults. The incidence of pertussis infections is on the rise, thus making it more important to vaccinate everyone who is a candidate. Sustained immunity to pertussis following the vaccine does not appear to be lifelong, necessitating at least 1 dose of Tdap after the age of 11.
• In cases of wounds requiring prophylaxis, a dose of the tetanus and diphtheria toxoid (Td) vaccine should be given, in case there is no documented history of Tdap vaccination previously. In addition, the Td vaccine should be routinely given every 10 years.
• Measles, mumps, and rubella (MMR) vaccine: Immunity rates to these diseases are high after just 1 dose of the vaccine and even higher after 2 doses.^51^ The vaccine is now routinely given to children. People who were born before 1957 are usually not vaccinated but can be assumed to have had natural infections and immunity. The immunity to these vaccines usually wanes somewhat with time, but protection from infection may be longer lasting than serologic conversion. The measles component of the vaccine is a live attenuated virus, whereas the other 2 components are recombinant vaccines. The MMR vaccine (and other live attenuated virus vaccines such as rotavirus, varicella, yellow fever, cholera, and smallpox) should therefore be avoided in patients receiving immunosuppression.

In cases of outbreaks of measles or rubella, 2 doses of MMR vaccines given at least 28 days apart should be given to those who are incompletely immunized. However, in cases of mumps outbreaks, a third dose of the MMR vaccine should be given to people who have already had 2 doses of the vaccine previously.

• One should follow recommendations for the general population for giving other vaccines to patients receiving dialysis, such as cholera, hepatitis A, hemophilus, papillomavirus, Japanese encephalitis virus, rotavirus, tick-borne encephalitis, meningococcal, rabies, typhoid, dengue, and yellow fever vaccines, until further data are available.^52^ Most of these vaccines are recommended for countries that have a significant burden of disease and are able to vaccinate large portions of their population. The hepatitis A vaccine may be the exception because the population in highly endemic countries develops disease in childhood, where the manifestations are minor but they develop long-lasting immunity. This vaccine is best given to population, at risk in areas of the world where hepatitis A is not endemic. In addition, BCG vaccination is recommended by the WHO in countries that have a high prevalence of *Mycobacterium tuberculosis* or *Mycobacterium leprae* infection. It is usually given during childhood, but adults should receive it too if not vaccinated. Materials on monkeypox vaccines do not specifically reference considerations for patients receiving dialysis, although the 2 available differ in that one is based on a live, replicating competent virus, thought to be problematic for immunocompromised hosts (Table 3).^53^

**Table 3:** Live and Inactivated Vaccines^48^

Live Vaccines^a^	Inactivated Vaccines
BCG Influenza (LAIV) MMR Varicella Yellow fever V. cholerae Typhoid 21a (oral) Smallpox/monkeypox (Jynneos)	*Haemophilus influenzae* Inactivated influenza Hepatitis A Hepatitis B Tetanus Pertussis Inactivated polio Pneumococcal Meningococcal Rabies Human papillomavirus Japanese encephalitis Traveler’s diarrhea and cholera (oral)

a Live vaccines are not appropriate for immunosuppressed patients.

## Conclusions

Vaccinating the patient receiving dialysis is of paramount importance because infection is a major cause of morbidity and mortality in this population, and observational data suggest a degree of protection from vaccines. Patients receiving dialysis are more likely to be diagnosed with serious infections, including influenza, SARS-CoV-2, and pneumonia. This reflects varying levels of immunocompromise and, potentially, frequent contacts with the health care system, often in a congregate setting. Furthermore, patients receiving dialysis are more likely to develop a more severe illness and require hospitalization, as was illustrated with the COVID-19 pandemic. The response to vaccination is not as strong as the general population, and rates of nonresponsiveness are significantly higher but the benefits of vaccination are also more pronounced because these patients are clearly at risk for worse outcomes if infected. Vaccinating in the dialysis center makes sense to increase the rates of vaccine uptake (Table 4 summarizes current recommendations).

**Table 4:** Current Recommendations for Vaccines Commonly Administered in Dialysis Facilities (Assuming no Previous Vaccination)

Disease	Vaccine Recommendation
**Pneumococcus**	PCV20 (1 dose)
**Influenza**	Fluzone (yearly if <65 y of age) Fluzone high dose (yearly if >65 y of age)
**Hepatitis B**	Heplisav-B (2 doses 1 mo apart)
**Severe acute respiratory syndrome coronavirus 2**	mRNA bivalent biannual booster shot after 2 baseline doses
